# Discovery of a Rodent Hepacivirus in the Brazilian Amazon

**DOI:** 10.3390/v17060830

**Published:** 2025-06-08

**Authors:** Nelielma G. Oliveira Prestes, Leonardo H. Almeida Hernández, Fábio Silva da Silva, Thito Y. Bezerra da Paz, Andressa O. Aragão, Bruno C. Veloso de Barros, Ricardo J. P. S. Guimarães, Rommel T. J. Ramos, Lívia Medeiros Neves Casseb, Sandro Patroca da Silva, Pedro Fernando da Costa Vasconcelos, Ana Cecília Ribeiro Cruz

**Affiliations:** 1 Department of Arbovirology and Hemorrhagic Fevers, Evandro Chagas Institute, Health and Environment Surveillance Secretariat, Ministry of Health, Ananindeua 67030-000, PA, Brazil; nelielmaprestes@gmail.com (N.G.O.P.); fabiosilva.analises@gmail.com (F.S.d.S.); thitodapaz000@gmail.com (T.Y.B.d.P.); liviacasseb@iec.gov.br (L.M.N.C.); spatroca@gmail.com (S.P.d.S.); pedro.vasconcelos@uepa.br (P.F.d.C.V.); anacecilia@iec.gov.br (A.C.R.C.); 2 Parasite Biology in the Amazon Region Graduate Program, Pará State University, Belém 66087-670, PA, Brazil; 3 Genomic and Systems Biology Center, Federal University of Pará, Belém 66075-110, PA, Brazil; andressa.arag@gmail.com (A.O.A.); rommelthiago@gmail.com (R.T.J.R.); 4 Faculdade Vale dos Carajás, Parauapebas 68515-000, PA, Brazil; brunocvb@yahoo.com.br; 5 Geoprocessing Laboratory, Evandro Chagas Institute, Health and Environment Surveillance Secretariat, Ministry of Health, Ananindeua 67030-000, PA, Brazil; ricardojpsg@gmail.com

**Keywords:** deforestation, hepacivirus, marsupialia, rodentia, virome

## Abstract

In the eastern Brazilian Amazon, the Viseu municipality has almost 70% of its territory deforested. Monitoring viruses from wildlife hosts enables the prevention and control of epizootic events and outbreaks. Seven samples from three marsupials and two rodents were screened by high-throughput sequencing for virome analysis. The three samples from the two *Proechimys roberti* rodents, one from the liver, one from the brain, and one from a pooled viscera sample, showed the highest results in terms of viral abundance and richness. From these we obtained two strains of a new rodent hepacivirus (RHV), which belongs to a new putative genotype of an unclassified RHV species previously described in Panama and Northeast Brazil. The findings expand the host range of the cited RHV species, imply virus circulation in the study area, and suggest a viral tropism in the liver and perhaps in the brain.

## 1. Introduction

Zoonotic diseases are a major public health concern due to their potential to emerge in human populations, in addition to their economic and environmental impacts. They depend on pathogens’ interaction with animals, which can act as vectors or hosts. This interaction, along with the consequent pathogen adaptation to new hosts, is facilitated by anthropogenic activities such as deforestation, climate change, and the exploitation of soil as well as natural resources. Among the emerging infectious diseases reported between 1940 and 2004, 60% were of a zoonotic origin, with most of them originating from wildlife animals [1,2].

The order Rodentia groups the largest number of species under the class Mammalia, and rodents are commonly associated as reservoirs of viruses. They may play a crucial role in the maintenance of viruses due to their high reproduction rates and wide distribution across forested and human-inhabited areas. Several RNA viruses are already known to be hosted by rodents, such as hantaviruses, arenaviruses, coronaviruses, paramyxoviruses, and flaviviruses [3,4,5].

The *Hepacivirus* genus belongs to the *Flaviviridae* family and currently includes 14 genetically diverse species of viruses, including the hepatitis C virus (HCV), known for its ability to infect and cause severe chronic liver disease in humans [6]. They have a linear positive-sense ssRNA genome that ranges in size from 8.9 to 10.5 kb and encodes three structural proteins—nucleocapsid core protein (C) and two envelope glycoproteins (E1 and E2)—an ion-channel-forming protein (p7), and six non-structural proteins (NS2, NS3, NS4A, NS4B, NS5A, and NS5B) [7].

Since 2011, with the discovery of homologous HCV in dogs, the genetic diversity of hepaciviruses has increased and new viruses have been discovered in other mammalian hosts, such as bats, horses, rodents, and even in other vertebrates. Despite the wide range of hosts, rodents and bats have been identified as the main transmitters to other mammals, since they harbor hepaciviruses with large genetic heterogeneity [8,9,10,11].

Monitoring viral species from samples obtained from wildlife hosts, whether in close contact with humans or not, enables the prevention and control of epizootic events, outbreaks, and even epidemics involving these viruses. To contribute to this effort, we applied a metagenomics approach to assess the diversity of RNA viruses in rodents and marsupials from Viseu, a municipality in eastern Brazilian Amazon (Pará State), which has 69.01% of its territory deforested [12].

## 2. Materials and Methods

### 2.1. Sample Collection

**Figure 1:**
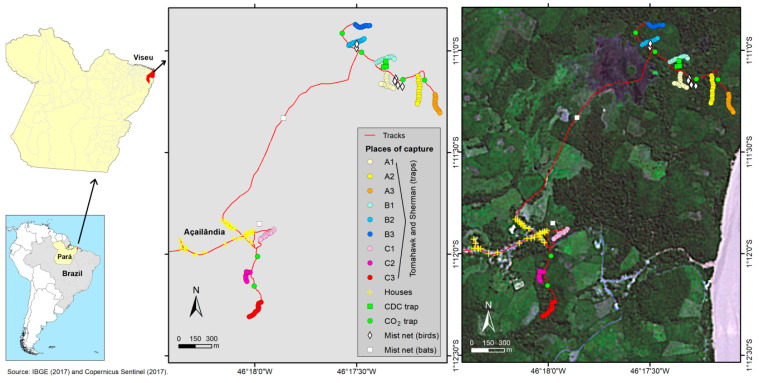
Location of capture sites in the Centro Alegre community, Viseu.

Biological samples from rodents and marsupials were collected during an eco-epidemiological investigation in the Centro Alegre community, in the municipality of Viseu, in the northeast of Pará State, Brazil (Figure 1) in November 2014 and August 2015. This study is part of a major virus surveillance project authorized by the Ethics Committee on Animal Use of the Evandro Chagas Institute (IEC) under certificate number 21/2014.

On the first expedition, three marsupials and one rodent were captured using live containment traps, including cage-type (Tomahawk) and aluminum (Sherman) traps. Two marsupials, MA7049 and MA7053, were captured near the village, at C2 and C3 places, respectively. The MA7054 marsupial and the RO22799 rodent were captured one mile away from the community, inside the forest fragment, at B2 and B1 places, respectively (Figure 1). The morphological identification of each animal was carried out prior to the collection of samples from multiple viscera. The procedure involved intramuscular sedation followed by euthanasia through an overdose of lidocaine administered into the foramen magnum. The animals were necropsied, and the harvested viscera were divided into two samples for each animal: one liver sample and a pooled viscera sample with fragments of the spleen, lymph nodes, heart, and lungs. From the second expedition, the RO22878 rodent was captured at an A1 place and euthanized using the same methods. Besides the liver and the pooled viscera samples, a brain sample was also collected. All of the eleven samples were stored in liquid nitrogen and transported to the Department of Arbovirology and Hemorrhagic Fevers of the IEC, where they were incubated at −80 °C.

### 2.2. RNA Extraction

A fragment of 5 mg of each sample was placed in a microtube and added to a 5 mm tungsten bead and 600 µL of 1-Thioglycerol/Homogenization Solution and homogenized using TissueLyser II equipment (Qiagen, Hilden, Germany) for 2 min at 25 Hz. Then, RNA extraction was performed using a Maxwell^®^ 16 LEV simplyRNA Tissue Kit (Promega, Madison, WI, USA) in a Maxwell^®^ 16 System (Promega), following the manufacturer’s protocol. The RNA concentration was evaluated using Qubit^®^ 2.0 Fluorometer equipment (Thermo Fischer Scientific, Waltham, MA, USA), and fragments’ size range was evaluated using a 2100 Bioanalyzer Instrument (Agilent Technologies, Santa Clara, CA, USA).

### 2.3. cDNA Synthesis, Library Preparation, and Sequencing

The first and second strands of complementary DNA (cDNA) were synthesized via SuperScript™ IV VILO™ MasterMix (Thermo Fischer Scientific) and NEBNext^®^ mRNA Second Strand Synthesis Module (New England BioLabs, Ipswich, MA, USA), respectively.

The cDNA library was prepared with a Nextera XT DNA Library Preparation kit (Illumina, San Diego, CA, USA), and its quantity and quality were assessed by using Qubit^®^ 2.0 Fluorometer equipment (Thermo Fischer Scientific) and a 2100 Bioanalyzer Instrument (Agilent Technologies), respectively. Finally, sequencing was performed on a NextSeq 500 System (Illumina) with a NextSeq 500/550 Mid Output kit (300 cycles), using the 150 bp paired-end methodology.

### 2.4. Bioinformatic Analysis

#### 2.4.1. Quality Control

Raw data were initially subjected to quality assessment using Fastp v.0.23.4 [13], configured to remove adapter sequences and reads with a base Phred quality score < 20 and a length of less than 50 nt. The removal of ribosomal reads was performed using SortMeRNA v.2.1 [14] based on the default database provided by the tool.

#### 2.4.2. Viral Diversity Assessment

After quality control, an assessment of the viral diversity at the family taxonomic level was carried out by using the DIAMOND tool v.2.1.9.163 [15] (Blastx, e-value of 10^−5^, taxonlist 10239) based on the non-redundant protein database (NR-NCBI, available at https://ftp.ncbi.nlm.nih.gov/blast/db/FASTA/, accessed on 12 December 2024). The obtained results were compiled into table files to calculate the general metrics of alpha diversity (heatmap of the relative abundance and diversity indices) using the R language [16], along with the ggplot2, pheatmap, and reshapet2 libraries.

#### 2.4.3. Sequence Assembly and Contig Inspection

The filtered reads were De Novo assembled using MEGAHIT v.1.2.9 software [17] (k-mers from 21 to 141). The obtained contigs were also compared against the NR protein database through DIAMOND v.2.1.9.163. Geneious Prime 2024.0 software [18] was used to inspect the generated contigs, and functional domain annotation was performed using InterPro v.101.0 [19].

#### 2.4.4. Phylogenetic Analysis

Analysis of the identified hepaciviruses was carried out using their obtained sequences along with closely related sequences available in the GenBank (NCBI) database. The sequences were initially aligned using the MAFFT v.7.520 algorithm [20]. Phylogenetic signal estimation and phylogenetic tree reconstructions based on the Maximum Likelihood method with bootstrap (BS) values set for 1000 replications were performed by IQ-TREE v.2.2.5 [21] for the polyprotein, the NS3 region, and the NS5B region. The resulting trees were visualized at Figtree v.1.4.496 [22] and edited using Inkscape v.0.92 [23]. Additionally, an amino acid p-distance analysis for the NS3 and NS5B regions was carried out by MEGA v.10.2.6 [24].

## 3. Results

### 3.1. Data Processing and Viral Diversity

Seven of the eleven samples were eligible for sequencing. Approximately 231.8 million reads were obtained from them, and only 16.7 million (~7.21%) were validated for genome assembly after quality control and ribosomal RNA removal (Table 1). The morphological identification of each animal was confirmed by the analysis of the *CytB* subunit of their mtDNA (Table S1).

**Figure 2:**
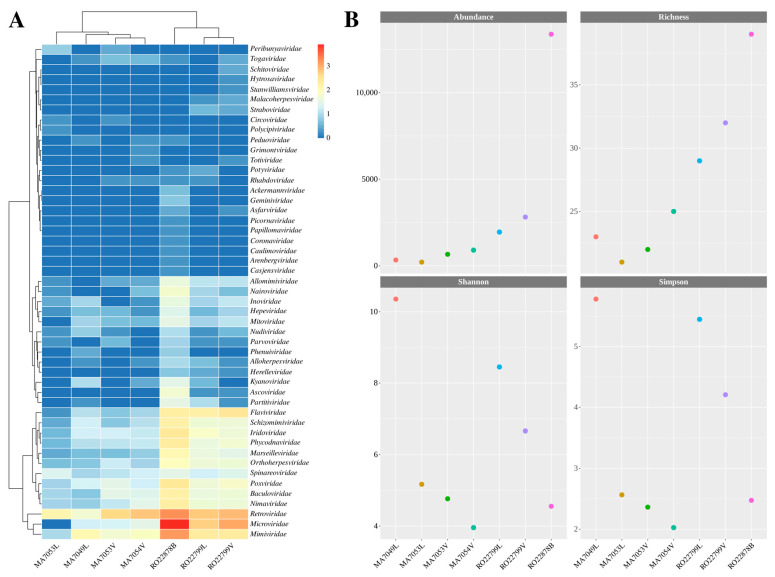
(**A**) Heatmap of viral reads on a normalized scale (log10) for the 49 viral families detected, distributed across the evaluated samples. The clusters indicate groupings based on abundance levels. The evaluated samples are displayed on the x-axis, and the potentially detected viral families are represented on the y-axis. (**B**) Alpha diversity graphs illustrate the observed metrics of abundance, richness, and Shannon as well as Simpson diversity indices.

As for the metrics regarding the abundance and diversity of viral reads, matches were recorded for 49 viral families, totaling 20,267 classified reads (Table S2). The most abundant viral families in terms of read classification were *Microviridae*, *Retroviridae*, *Mimiviridae,* and *Flaviviridae* (Figure 2A), accounting for 83.3% of the classified viral reads. The RO22878B (*Proechimys roberti*) showed the highest results in terms of abundance (classified viral reads) and richness (diversity of classified viral families) compared to the other samples (Figure 2B). However, analysis of the Shannon and Simpson diversity indices indicated that the MA7049L (*Marmosa murina*) and RO22799L samples had higher diversity (Figure 2B, Table S3).

### 3.2. Inspection of Hepacivirus Sequences

Viral contigs identified in both RO22799 samples and in the RO22878B sample have been shown to be close to two rodent hepacivirus (RHV) sequences: MG822666 (Panama) and PP840897 (Northeast Brazil, Cavia hepacivirus—CHV). A complete sequence of 8372 nt was recovered from the RO22799L sample (MW417366), while a partial sequence of 8265 nt, only covering the viral polyprotein ORF and containing three gaps, was obtained from the RO22799V sample (PV112702). Furthermore, the sequence from the RO22878B sample (MW417365) has a length of 8415 nt, considering the four gaps, and is incomplete at the polyprotein 3′ end.

**Figure 3:**
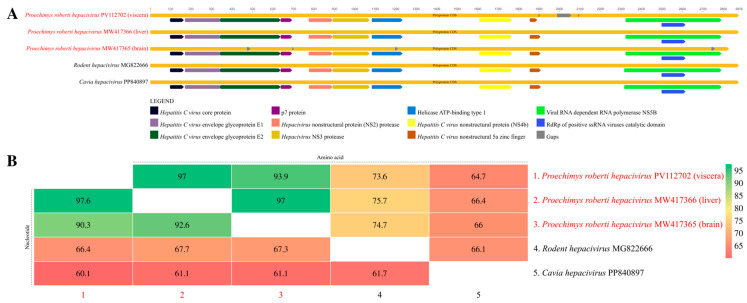
(**A**) Representation of the *Hepacivirus* polyprotein functional domains, comparing the identified contigs with close sequences. (**B**) Heatmap of the polyprotein’s nucleotide (lower triangle) and amino acid (upper triangle) identities of the three study sequences and close sequences.

The three sequences presented functional domains associated with the *Hepacivirus* genus (Figure 3A), like the reference sequences. When the entire polyprotein is considered, the RO22799 sequences showed 97.6% and 97% of nucleotide and amino acid identity, respectively, between each other, and an average of 91.4% and 95.4% of nucleotide and amino acid identity, respectively, with the sequence from the brain (Figure 3B).

Considering the NS3 region, which has no gaps in any of the sequences, both nucleotide and amino acid identities are 100% between the RO22799 sequences. These identities are reduced to 92.6% for the nucleotide and 97% for the amino acid analysis when both RO22799 samples are compared with the sequence from the brain. For the NS5B region, the nucleotide and amino acid identities are, respectively, 99.9% and 100% between the RO22799 sequences, and 92.45% and 96.5% between them and the RO22878B sequence (Figure S1).

**Figure 4:**
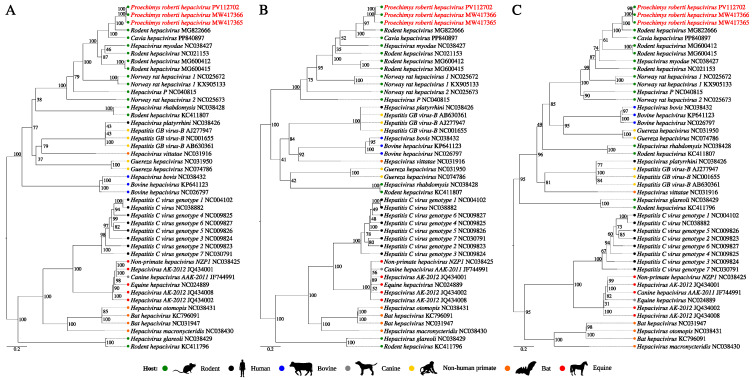
Phylogenetic reconstruction based on the amino acid sequences of the *Hepacivirus* (**A**) polyprotein region, (**B**) NS3 region, and (**C**) NS5B region. The obtained sequences are highlighted in red.

The phylogenetic reconstruction analysis based on 43 polyprotein nucleotide sequences from the *Hepacivirus* genus (Table S4), along with the three new sequences, resulted in a topology supported by high internal anchoring values (Figure 4A). The identified sequences were positioned together with the two previously cited RHV sequences (MG822666 and PP840897) (BS = 100%), within a clade composed exclusively of viruses isolated from rodents. Additionally, the obtained topology was supported by a phylogenetic signal of 97.9% (Figure S2).

The NS3 and NS5B regions are the most conserved for hepaciviruses and used for species demarcation criteria by the International Committee on Taxonomy of Viruses (ICTV). The topologies of the trees generated based on these genetic markers remained identical to the previous one and were also supported by high phylogenetic signals (Figure S2). In both trees, the three study sequences were grouped with the RHV from Panama as well as the CHV sequences (Figure 4B,C), with a high BS value. Considering the amino acid p-distances, the pairwise comparison between the five sequences presented values lower than the determined threshold for species demarcation: 0.25 for the NS3 region and 0.3 for the NS5B region (Tables S5–S7), which implies their classification under the same RHV species. These values were greater when they were compared with the other hepacivirus sequences.

## 4. Discussion

Zoonotic diseases have a great impact on public health, and their emergence is directly related to anthropogenic pressures. The Brazilian Amazon is currently facing several threats to its biodiversity, and deforestation plays a major role in wildlife closeness with human communities. Although not every spillover event involves humans, the very ability of the pathogen to adapt to a new host represents a warning for public health [25,26]. In this context, understanding the evolution, origin, and circulation dynamics of animal viruses—both novel and previously described—can provide information with which to predict viral emergence.

Virome analysis shows us the diversity of viruses presented in a host or ecosystem and the relationship with the diversity of other research subjects. Even though the viral community varies in time and space, the metagenomic approach allows diversity analysis and comparison, considering these biases, and the discovery as well as characterization of viruses that would not be identified by isolation and not included in common PCR screenings [27,28].

In this study, we analyzed seven samples from five different hosts. Four samples were from marsupials—two of which were liver samples and two were pooled viscera samples—and three samples from rodents—one sample from the liver, one from the brain, and one pooled viscera sample. Diversity analysis based on the classification of reads from the evaluated samples showed higher abundances for the viral families *Retroviridae* and *Mimiviridae* in all samples, for the *Microviridae* family in six samples, and for the *Flaviviridae* family in the three rodent samples.

The *Retroviridae* family is commonly identified in samples from metagenomic studies in small mammals and can represent the existence of endogenous retroviruses integrated into host genomes [29,30,31,32]. On the other hand, the *Microviridae* family groups DNA phages present in different environments and hosts and the *Mimiviridae* viruses supposedly infect amoebae, and their identification could be related to environmental contamination [33,34]. Lastly, the higher abundance of *Flaviviridae* reads in the rodent samples is directly related to the detection of hepacivirus strains.

When analyzed, the RO22878B sample had the highest rates of abundance and richness, indicating a higher frequency of viral reads and the number of viral families they were classified into, which can be observed on the heatmap representation. The Shannon and Simpson diversity indices, on the other hand, were higher for MA7049L and both RO22799 samples and only then for the RO22878B sample, which is associated with a better distribution of classified reads across the represented viral families in comparison to the RO22878B, for which more reads were assigned to the *Microviridae* and *Retroviridae* families.

The discovery of numerous animal hepaciviruses over the last fourteen years is helping to elucidate the possible origin of HCV, which is now suggested to be the result of cross-species transmission. RHVs have the greatest genetic divergence among hepaciviruses, even diverging from one another, with lineages positioning in different phylogenetic clades [6,35,36]. Furthermore, a recent study demonstrated that RHVs diverged earlier than other mammalian hepaciviruses, which may have mostly originated from rodents [11].

This is the first description of a RHV in the Brazilian Amazon and stands out for its detection in tissue samples. In contrast, sequences from other RHVs from Latin America countries—Panama and Brazil—were obtained from blood or sera samples [37,38,39]. From the same rodent, a hepacivirus was detected in the liver and in the pooled viscera sample. Moreover, another sequence was obtained from a brain sample of another rodent, an unexpected finding. Even though complementary tests would be required to investigate the viral ability to infect each organ, the hepatic tropism was already described for some RHVs [35,40] and the neurotropism was suggested for *Hepacivirus equi* [41,42].

The three sequences share the same functional domain arrangement as the other two RHV sequences and their proximity is corroborated in the phylogenetic analysis, where they clustered in the same clade with a well-supported BS value. Although the CHV (Northeast Brazil) was identified in a Caviidae rodent, the RHV from Panama and the three sequences from this study were identified in *Proechimys* (Echimyidae) rodents. The rodent from Panama was a *P. semispinosus*, while the RO22799 and RO22878 rodents were from the *P*. *roberti* species, which is distributed in the eastern Amazon basin and Cerrado biome, being the only *Proechymis* species with distribution reported in the study area [43,44,45] and was already described as hosting some parasites and *Mycobacterium leprae* [46,47,48].

The species demarcation criteria for the *Hepacivirus* genus determine that amino acid p-distances greater than 0.25 and 0.3 for the NS3 and NS5B regions, respectively, must be considered to distinguish species of hepaciviruses [6]. The pairwise comparison between the five sequences showed values below the stipulated thresholds, which was already observed between the RHV from Panama and the CHV, suggested to be two different genotypes from the same putative species [39]. It is noteworthy that the sequences obtained from different sample types from the same rodent are the same strain, but the sequence from the brain is slightly different, when the p-distances and nucleotide as well as amino acid identities for the complete polyprotein and NS3/NS5B regions are considered, which suggests the accumulation of mutations within the same genotype. However, the genetic divergence is less than 10% at the nt level, which could not be considered a different genotype or even a subtype, following the same demarcation point for the HCV [6]. Therefore, the discovery of two strains of a virus, tentatively named as Proechimys roberti hepacivirus (PrHV), from a putative new genotype for the unclassified RHV species under discussion is suggested.

Rodents are one of the main transmitters of hepaciviruses, and some RHVs have already been shown to have potential zoonotic relevance [49]. The two rodents with hepaciviruses were captured inside the forest fragment, one mile away from the community, and wild rodents often circulate outside their natural habitats due to anthropic activities, as found in the area represented in this study. This is a scenario that demands constant monitoring for viral circulation due to the possibility of events of public health interest.

## 5. Conclusions

This study provides a virome analysis of five animals from a deforested area in the eastern Brazilian Amazon. Among the viral contigs identified, it was possible to discover PrHV, the first RHV from the Brazilian Amazon, corroborating a wider distribution of RHVs in South America. The two strains of PrHV, one from 2014 and another from 2015, belong to a putative new genotype of an unclassified RHV species. Furthermore, the findings expand the host range to another *Proechimys* species and imply virus circulation in the study area. In addition, they suggest viral tropism in the liver and provide a report of PrHV in a brain sample. Its unknown zoonotic potential, pathogenesis, and prevalence among rodent populations highlight the need for more analyses and sample collections in the region.

## Figures and Tables

**Table 1:** Sample and raw data processing information.

Sample ID	Host Species	Collection Date	Total Reads	Fastp	SortMeRNA	Removed	Validated (%)
MA7049L ^1^	*Marmosa murina*	27 November 2014	23,083,368	20,080,020	1,155,010	21,928,358	5.0
MA7053L	*Philander* *opossum*	27 November 2014	34,585,464	30,206,256	1,052,840	33,532,624	3.0
MA7053V ^2^			38,751,852	34,305,968	2,507,726	36,244,126	6.5
MA7054V	*M. demerarae*	27 November 2014	33,483,380	29,036,538	6,645,268	26,838,112	19.8
RO22799L	*Proechimys* *roberti*	28 November 2014	29,624,298	25,665,772	927,080	28,697,218	3.1
RO22799V			38,632,322	33,938,920	1,602,564	37,029,758	4.1
RO22878B ^3^	*P. roberti*	20 August 2015	33,642,686	29,172,270	3,652,558	29,990,128	10.9

^1^ L for liver sample; ^2^ V for viscera sample; and ^3^ B for brain sample.

## Data Availability

The study sequences were deposited in GenBank under the accession numbers MW417365, MW417366, and PV112702, and the raw sequencing data have the following SRA numbers: SRR32311131 to SRR32311136 and SRR32463401. The CytB sequences were deposited under accession numbers PV125361 to PV125366 and PV173300.

## Supplementary Materials

The following supporting information can be downloaded at: https://www.mdpi.com/article/10.3390/v17060830/s1, Figure S1: Nucleotide and amino acid identity matrix of the (A) NS3 and (B) NS5B regions; Figure S2: Phylogenetic signal mapping diagrams showing the quartet analysis for the (A) polyprotein, (B) NS3, and (C) NS5B reconstructed trees; Table S1: Results of *CytB* analysis, Table S2: Viral read classification by viral family and number of reads; Table S3: Abundance, richness, and Shannon as well as Simpson diversity indices metric analysis; Table S4: Sequences used in the evolutionary analysis; Table S5: Amino acid p-distance calculated based on the polyprotein; Table S6: Amino acid p-distance calculated based on the NS3 region; Table S7: Amino acid p-distance calculated based on the NS5B region.
