# Supplementary material for: Discovery of a Rodent Hepacivirus in the Brazilian Amazon
Source: Viruses. 2025 Jun 8;17(6):830. doi: 10.3390/v17060830 (PMC12197313; doi:10.3390/v17060830)
Supplement: Supplementary file 1 [file viruses-17-00830-s001.zip › Supplementary Figures.pdf]

Supplementary Figures

NS3 protein

Upper triangle: Amino acid; lower triangle: Nucleotide

	1	2	3	4	5	
1		100	97	82	76.8	1. *Proechimys roberti hepacivirus* PV112702 (viscera)
2	100		97	82	76.8	2. *Proechimys roberti hepacivirus* MW417366 (liver)
3	92.6	92.6		79.8	75.2	3. *Proechimys roberti hepacivirus* MW417365 (brain)
4	68.4	68.4	67.9		77	4. *Rodent hepacivirus* MG822666
5	66.9	66.9	66.4	68.3		5. *Cavia hepacivirus* PP840897

Colour scale: 100, 95, 90, 85, 80, 75, 70

NS5B protein

Upper triangle: Amino acid; lower triangle: Nucleotide

	1	2	3	4	5	
1		100	96.5	81.5	72	1. *Proechimys roberti hepacivirus* PV112702 (viscera)
2	99.9		96.5	81.5	72	2. *Proechimys roberti hepacivirus* MW417366 (liver)
3	92.4	92.5		81.7	74	3. *Proechimys roberti hepacivirus* MW417365 (brain)
4	71.7	71.7	71.2		73.2	4. *Rodent hepacivirus* MG822666
5	65	65	65.2	67.1		5. *Cavia hepacivirus* PP840897

Colour scale: 100, 95, 90, 85, 80, 75, 70

Figure S1. Nucleotide and amino acid identity matrix of the (A) NS3 and (B) NS5B regions.

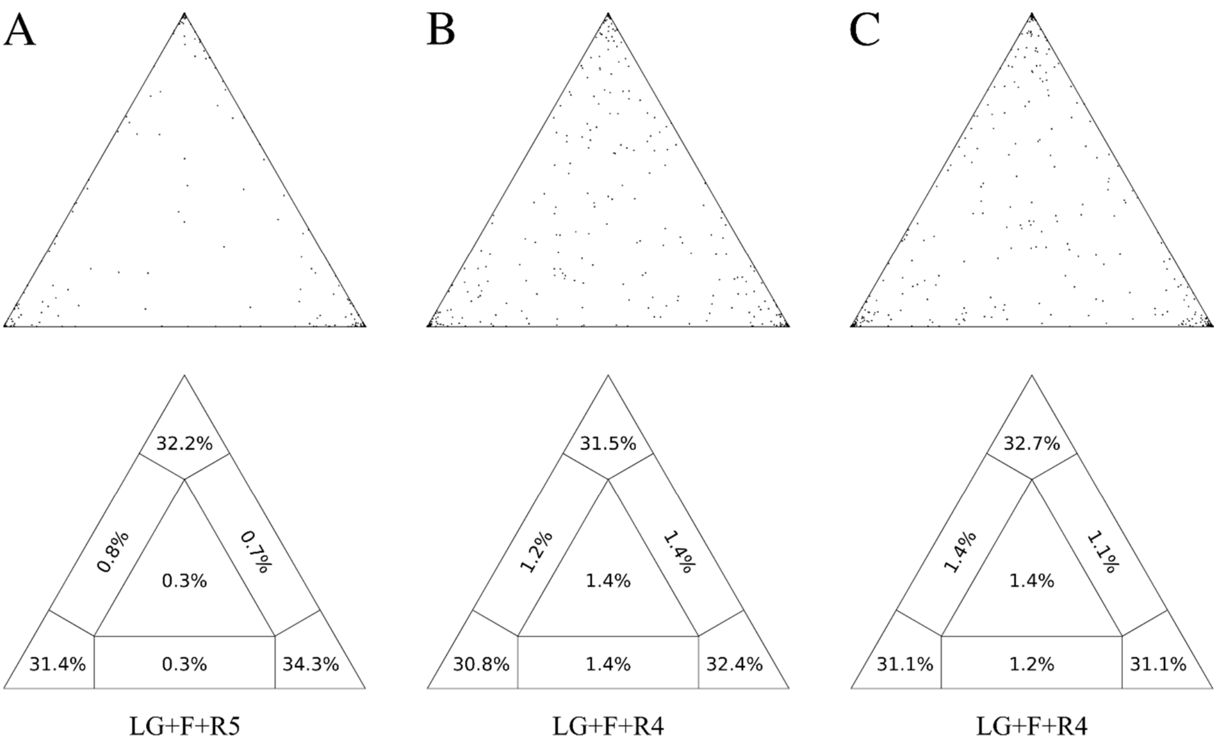

Figure S2. Phylogenetic signal mapping diagrams showing the quartet analysis for the (A) polyprotein, (B) NS3, and (C) NS5B reconstructed trees.
